# Is there any association between *Toxoplasma gondii* infection and depression? A systematic review and meta-analysis

**DOI:** 10.1371/journal.pone.0218524

**Published:** 2019-06-13

**Authors:** Tooran Nayeri Chegeni, Mehdi Sharif, Shahabeddin Sarvi, Mahmood Moosazadeh, Mahbobeh Montazeri, Sargis A. Aghayan, Nader Jafari Balalami, Shirzad Gholami, Zahra Hosseininejad, Reza Saberi, Davood Anvari, Shaban Gohardehi, Ahmad Daryani

**Affiliations:** 1 Toxoplasmosis Research Center, Mazandaran University of Medical Sciences, Sari, Iran; 2 Department of Parasitology, School of Medicine, Mazandaran University of Medical Sciences, Sari, Iran; 3 Student Research Committee, Mazandaran University of Medical Sciences, Sari, Iran; 4 Health Sciences Research Center, Addiction Institute, Mazandaran University of Medical Sciences, Sari, Iran; 5 Laboratory of Zoology, Research Institute of Biology, Yerevan State University, Yerevan, Armenia; 6 Department of Humanity and Social Science, Mazandaran University, Babolsar, Iran; Semmelweis University, HUNGARY

## Abstract

**Background:**

*Toxoplasma gondii* (*T*. *gondii*) is an obligate intracellular opportunistic parasite that is the causative agent of toxoplasmosis. This parasite accounts for mental disorders; however, the relationship between *T*. *gondii* infection and depressive disorder is unclear. Regarding this, the present systematic review and meta-analysis was conducted to investigate the scientific evidence regarding the potential association between major depression disorder (MDD) and *Toxoplasma* infection.

**Methods:**

For the purpose of the study, the articles related to the subject of interest were systematically searched in seven electronic databases. Special attention was given to the studies examining *T*. *gondii* seropositivity level in depressed patients and controls.

**Results:**

The search process resulted in the identification of a total of 30 publications meeting the inclusion criteria and published up to April 2018 for the systematic review. Furthermore, 29 studies met the inclusion criteria to be entered into meta-analysis. Our meta-analysis involved the review of cross-sectional studies including 1657 depressed patients and 19565 individuals as controls and case-control studies entailing 1311 depressed cases and 6015 controls without depression. 1582 depressed people participated in cross-sectional studies whose results were reported as odds ratio (OR). In addition, the total number of participants was 15068 in this type of studies. Statistical analysis indicated that the pooled OR of the risk of anti-*T*. *gondii* IgG antibody in depressed individuals in case-control and cross-sectional studies was 1.15 (95% confidence interval (CI): 0.95–1.39).

**Conclusions:**

As the findings of the reviewed articles indicated, toxoplasmosis is not a risk factor for MDD. However, it is necessary to perform further research to clarify the detailed association between *T*. *gondii* and dysthymia or mild and moderate depression. Furthermore, it is recommended to better investigate the effect of antibody titers on the relationship between depression and *T*. *gondii* infection.

## Introduction

*Toxoplasma gondii* (*T*. *gondii*) is an obligate neurotropic protozoan parasite that forms cysts in some tissues, including the brain of warm-blooded mammals like humans [1]. Cats are the final hosts of this parasite, and human infection occurs often via the ingestion of oocyst in water or tissue cyst in undercooked or raw meat [2]. *Toxoplasma gondii* infects about 25–30% of the people in developed and developing countries [1]. Most of *T*. *gondii* infections in immunocompetent individuals are asymptomatic. Nevertheless, in congenital disorders and immunocompromised patients, the infection may lead to the eye, lymph node, and central nervous system diseases [1, 3, 4].

The neurotropic nature and other specifications of *T*. *gondii* have made it a potential causative agent for psychiatric and behavioral disorders. *T*. *gondii* uses a complicated mechanism to gain access to the brain. When *T*. *gondii* reaches the brain, it invades different brain cells, including astrocytes and neurons, where it forms cysts [5]. According to the evidence, latent toxoplasmosis causes behavioral disorders not only in mice but also in humans [6, 7].

Recently published systematic review articles have proven the relationship between *T*. *gondii* infection and some psychiatric disorders such as bipolar disorder [8, 9], schizophrenia [9, 10], and epilepsy [11]. Among behavioral disorders, depression as the most common mental disorder, is coupled with remarkable morbidity and mortality. According to the Diagnostic and Statistical Manual of Mental Disorder (DSM-V) criteria, major depressive disorder (MDD) is a mental disorder characterized by at least two weeks of feeling low mood and disappointment. It is often accompanied by decreased self-esteem, loss of interest, cognitive performance, delight, dream, appetite, energy level, feelings of worthlessness, changes in weight, and having suicidal ideation and attempt for suicide [12–14].

So far, two systematic reviews have evaluated the relationship between toxoplasmosis and depression. One study has indicated that depression might be associated with microbial infections, such as those caused by *T*. *gondii*, human herpesvirus, hepatitis B virus, Chlamydiaceae, and Borna disease virus [15]. In addition, in a recent systematic review, several psychiatric disorders, namely schizophrenia, bipolar disorder, obsessive-compulsive disorder, and addiction, have been reported to be in association with toxoplasmosis. However, in the mentioned study, no significant relationship was found between depression and toxoplasmosis [9]. Since the results of the available original articles are contradictory, the aim of this new systematic review was to comprehensively assess the association between MDD and toxoplasmosis adding the latest studies on this matter. This study was also targeted toward the investigation of the susceptibility of toxoplasmosis patients to depression.

## Methods

### Design and protocol registration

The preferred reporting items for systematic reviews and meta-analysis (PRISMA) was used for performing this study (S1 Table) [16]. The study protocol (CRD42017069169) was registered on the site of the international prospective register of systematic reviews (PROSPERO) [17].

### Search strategy

For the purpose of the study, the original studies investigating the association between toxoplasmosis and depression and published up to April 2018 were systematically searched in several databases, including “PubMed”, “Science Direct”, “Scopus”, “ProQuest”, “EMBASE”, “Web of Science”, and “Google Scholar” with no language restriction. The search process was accomplished using the following keywords in combination or alone: “*Toxoplasma gondii*”, “Toxoplasmosis”, “Prevalence”, “Seroprevalence”, “Depression”, “Depressed”, “Depressive”, “Psychic disorder”, “Mental disorder”, “Systematic review”, and “Meta-analysis”. In order to achieve additional eligible articles, the reference lists of the retrieved articles were checked. In addition, unpublished studies were not investigated in the review process.

### Inclusion and exclusion criteria

We included articles based on the following criteria: 1) cross-sectional and case-control studies investigating the relationship between toxoplasmosis and MDD, 2) the studies performed only on humans, 3) original articles with available full texts in all languages, and 4) studies providing detailed information on the prevalence of toxoplasmosis using serology (i.e., IgG and/or IgM antibodies) and molecular tests in people with the diagnosis of depression according to the DSM-5 criteria [12]. On the other hand, the exclusion criteria entailed: 1) studies not representative for our target population, 2) studies performed on animal models; 3) low-quality data, 4) case reports or case series, and 5) studies with incomplete data.

### Study selection and data extraction

All identified titles and abstracts were independently assessed for eligibility by two authors using a pilot form. Any disagreements in the selected studies were resolved by discussion, and the arbitration of the third author. The included papers were attentively studied, and the information about the study location, number and percentage of the positive and negative cases of toxoplasmosis in patients and controls, number and percentage of depressed patients and controls, age, gender, method of studies, all laboratory results, and Odds ratio (OR) were extracted using a data extraction form.

### Quality assessment

Assessment of methodological quality was performed by two investigators using the Newcastle-Ottawa Scale [18] with separate criteria for case-control and cross-sectional studies to evaluate the selection, comparability, and outcome of the included studies. In the case-control studies, the scores of 7–9, 4–6, and ≤ 3 were representative of high, moderate, and low quality, respectively. Furthermore, regarding the cross-sectional studies, the scores of 6–7, 3–5, and 1–2 indicated high, moderate, and low quality, respectively. In this regard, the higher scores were awarded to the studies of higher quality. The quality of the included studies is reported in S2 Table.

### Statistical analysis

The meta-analysis was performed to evaluate the potential for the association between *T*. *gondii* infection and MDD using Stata software, version 14 (StataCorp, College Station, TX, USA) [19]. The heterogeneity index among different studies was determined using Cochran’s Q test [19]. The ORs of the risk of anti-*T*. *gondii* IgG and IgM antibodies in depressed patients were estimated using a random effects model. The relationship between toxoplasmosis and MDD was expressed as OR for seven cross-sectional studies. For the other five cross-sectional studies presenting results as number and percentage, the OR was calculated. In addition, for the 17 case-control studies not reporting the association between toxoplasmosis and depression as OR, we calculated OR and 95% confidence interval (CI) using the raw number of individuals in case and control groups. Finally, 29 articles were analyzed. An OR > 1 indicates the positive effect of *Toxoplasma* on MDD, and an OR < 1 shows that toxoplasmosis has a protective effect against MDD.

A funnel plot and Egger’s regression test were used to check for the presence of publication bias, and the significance level was less than 0.1 [20]. Furthermore, a sensitivity analysis was performed to identify the effect of each study on the overall results through removing each study. The subgroup analysis was conducted according to the type of the study. Also, analysis of the effect of study quality on the overall effect was performed.

## Results

**Fig 1:**
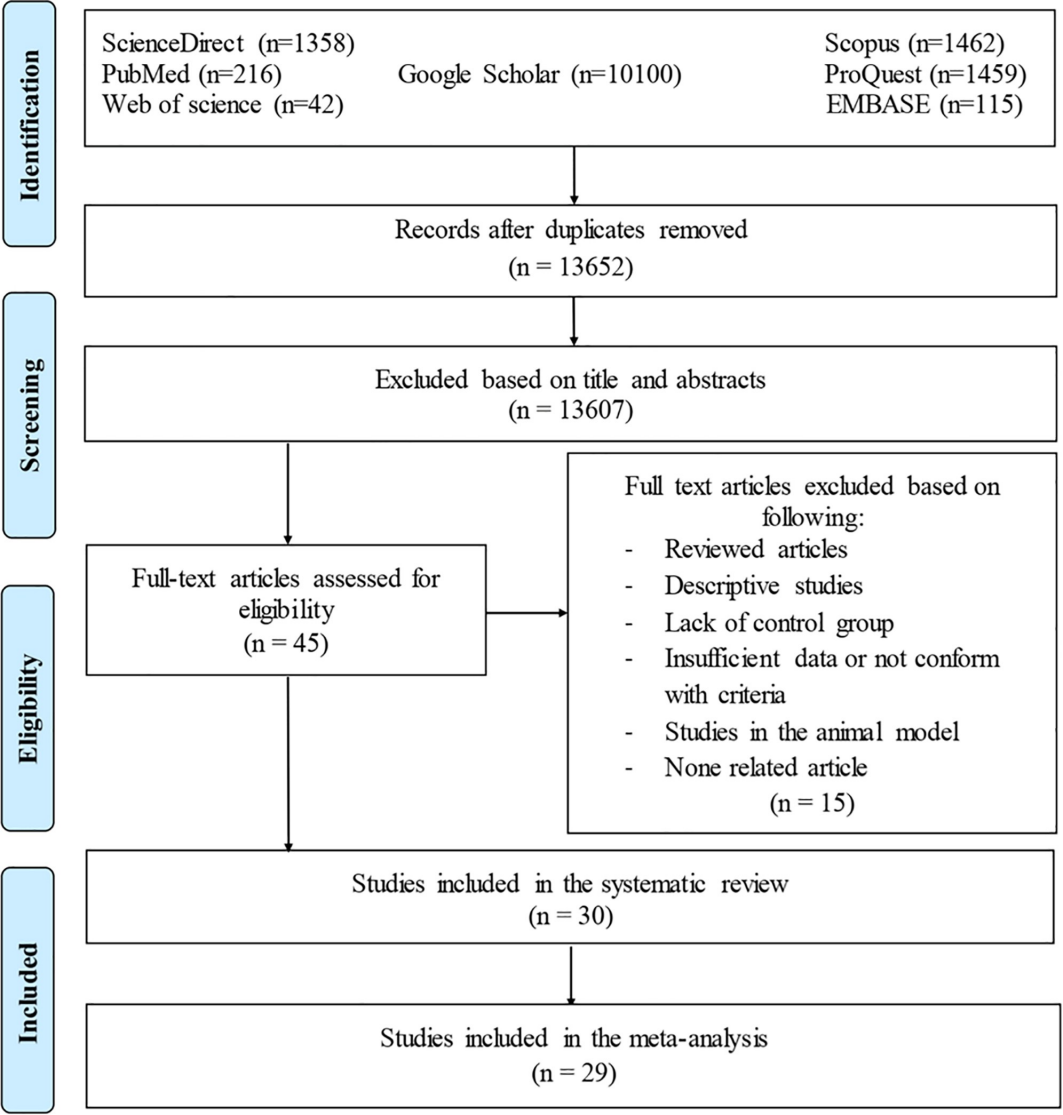
The PRISMA flow diagram of the search strategy, study selection, and data management procedure of *T*. *gondii* infection and depression.

As shown in Fig 1, the literature search resulted in the identification of 13,606 related articles. After eliminating the duplicate publications, a total of 12,587 articles remained. Following a thorough examination of the titles and abstracts of the papers, a total of 45 studies were extracted. Finally, out of 45 unique records, 30 articles were eligible to be included in this systematic review, 29 cases of which met our inclusion criteria for meta-analysis. A study conducted by Groër was not analyzed due to the lack of a control group [21].

**Fig 2:**
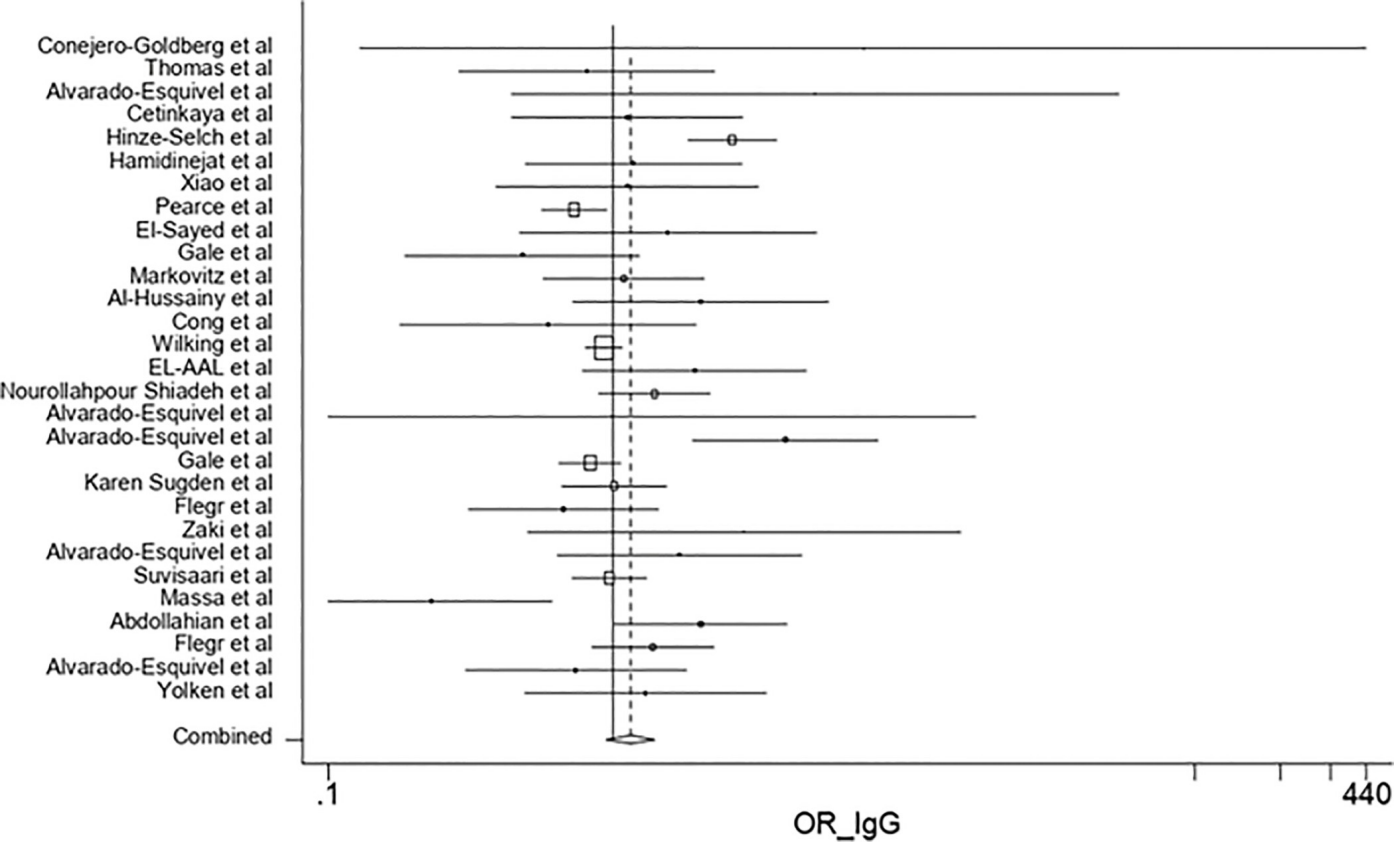
Forest plot diagram of studies showing IgG seropositivity rates of *T*. *gondii*. Perpendicular discontinuous line indicates the odds ratio index. The perpendicular continuous line represents the null hypothesis. The horizontal lines illustrate 95% CI for ORs. A study that its confidence interval (horizontal line) interrupts the vertical continuous line (line null hypothesis), the odd ratio of this study is not statistically significant.

**Fig 3:**
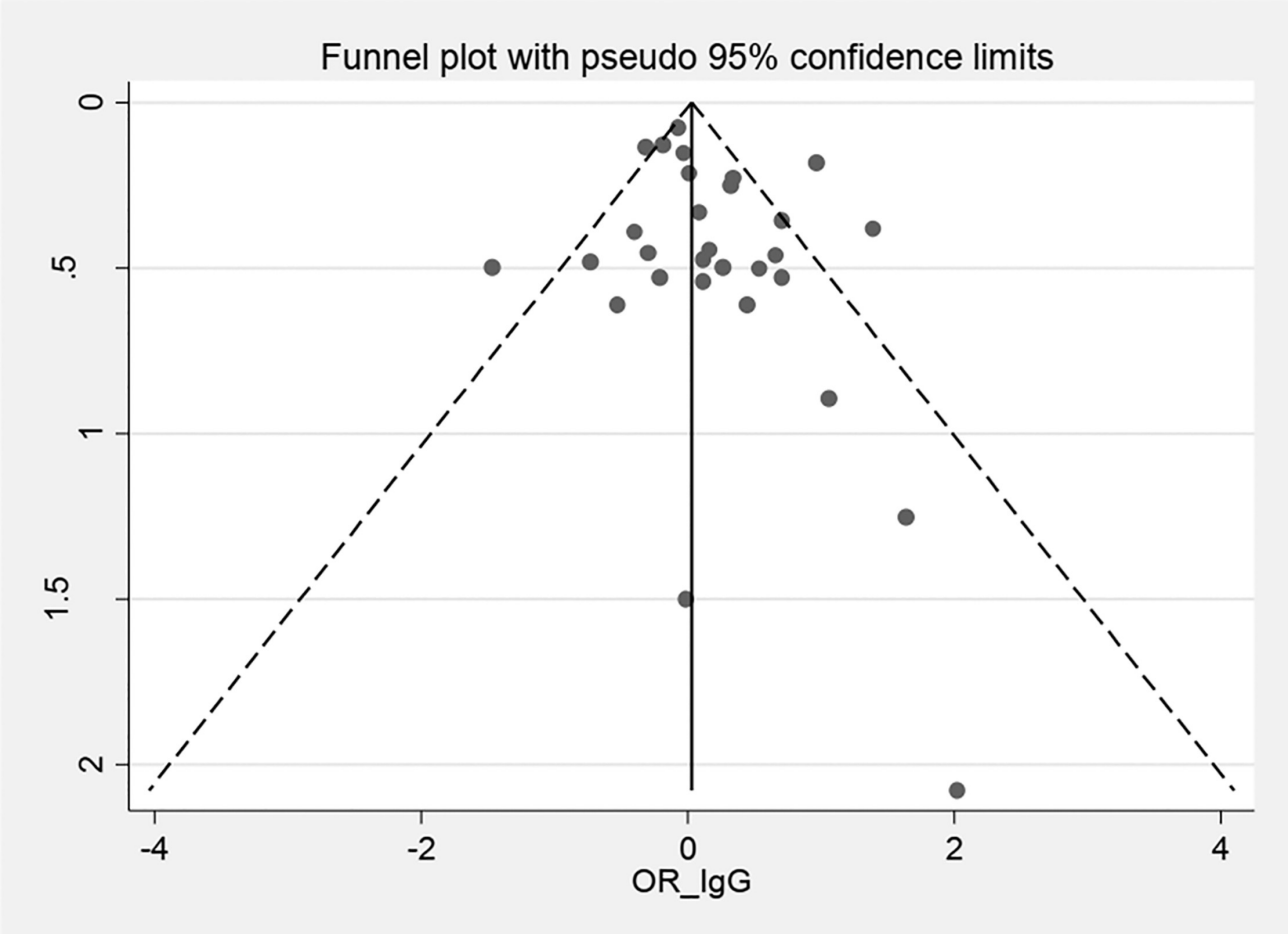
A bias assessment plot from Egger.

According to the results of the meta-analysis, the pooled OR of the risk of anti-*T*. *gondii* IgG antibody in the depressed subjects investigated in the included studies was 1.15 (95% CI: 0.95–1.39) (Fig 2). The test of heterogeneity revealed a significant heterogeneity among the studies (Q = 80.58%, *p* = 0.000). Publication bias was evaluated using the Egger's test (*p* = 0.148). On the other hands, as funnel plot shows the graph is symmetric and there is no publication bias (Fig 3). Furthermore, the OR of the risk of anti-*T*. *gondii* IgM antibody in depressed individuals was estimated at 1.69 (95% CI: 0.72–3.96).

**Fig 4:**
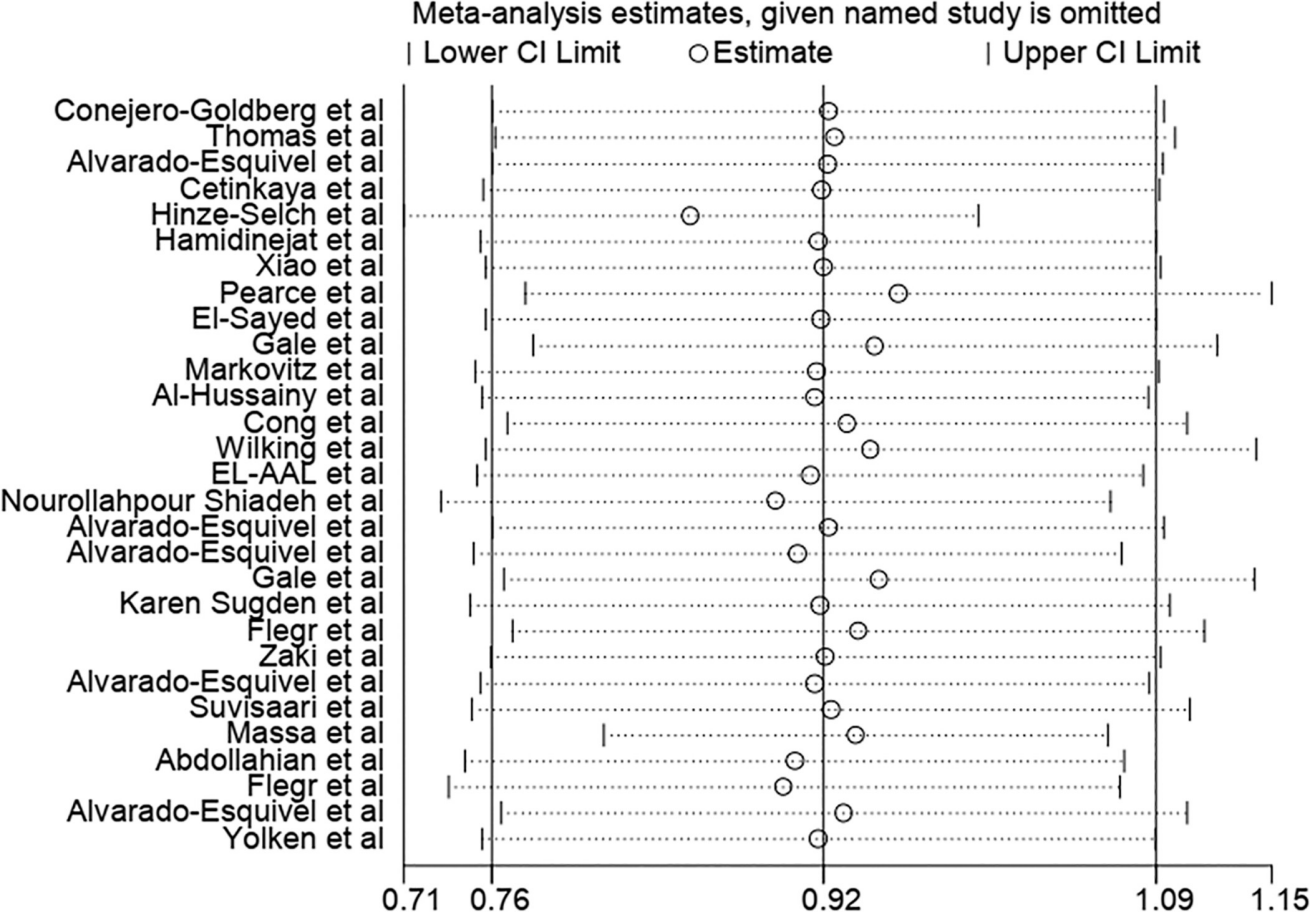
Sensitivity analysis for assessing the effect of each primary study on the total estimates.

**Fig 5:**
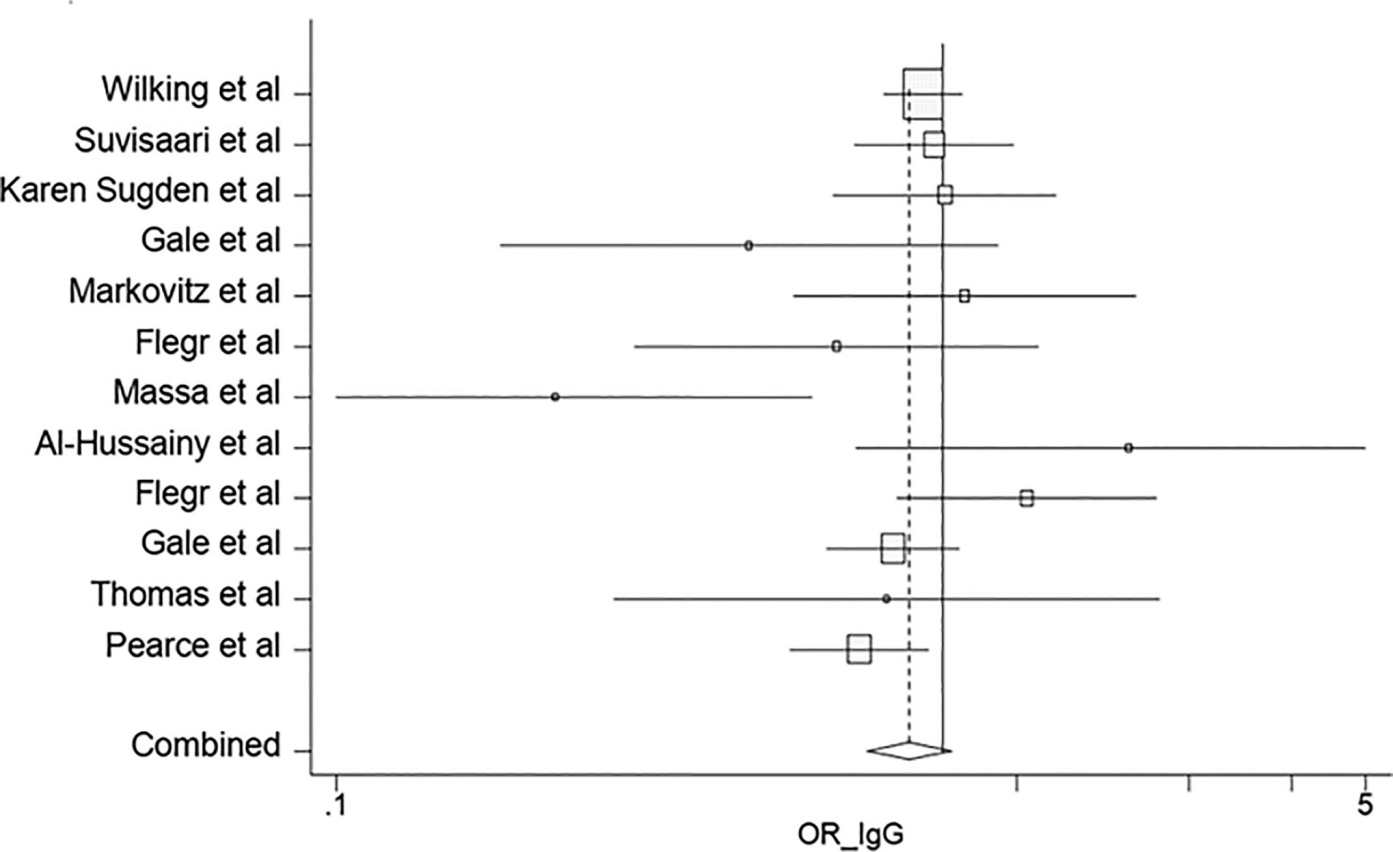
Forest plot diagram of cross-sectional studies showing IgG seropositivity rates of *T*. *gondii*.

**Fig 6:**
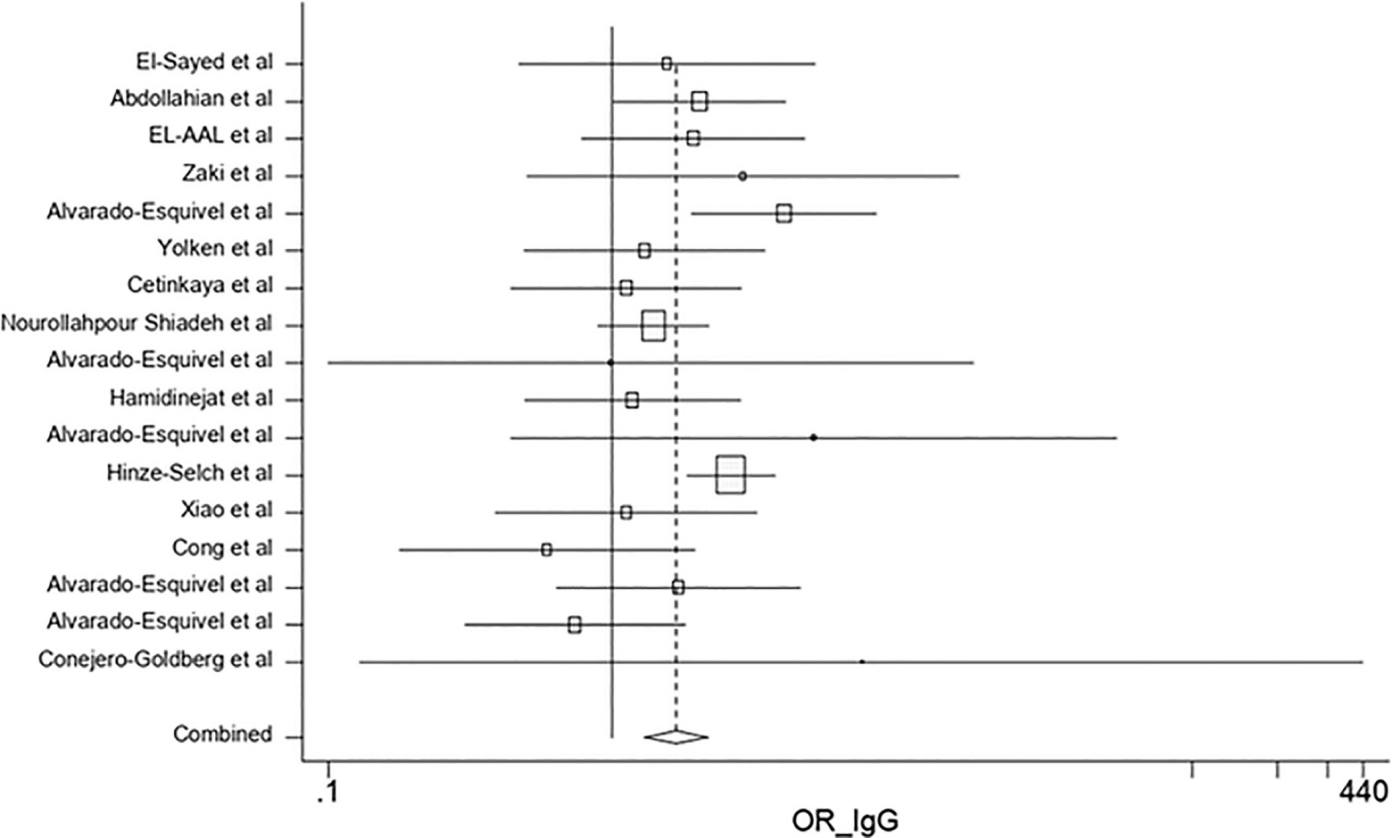
Forest plot diagram of case-control studies showing IgG seropositivity rates of *T*. *gondii*.

The results of the sensitivity analysis demonstrated that the impact of each study on overall estimates was not significant (Fig 4). According to the random effects model, the combined ORs of the risk of anti-*T*. *gondii* IgG antibody in depressed subjects based on the type of research in cross-sectional and case-control studies were 0.88 (95% CI: 0.75–1.03) and 1.67 (95% CI: 1.29–2.16), respectively (Figs 5 and 6). The quality assessment scores were evaluated as effective factors in overall estimation using meta regression; it has been shown that the impact of quality of each study on the overall estimates was not statistically significant (p = 0.275).

A total of 1657 patients with MDD and 19565 controls in cross-sectional studies and 1311 depressed cases and 6015 controls in case-control studies were included in the meta-analysis. Furthermore, 1582 depressed people participated in cross-sectional studies and their results were reported as OR. The total number of participants in this type of study was 15068.

Information and characteristics of the included publications are shown in Tables 1 and 2. The studies reviewed in this research were from 13 countries. Most of the studies were conducted in the USA (n = 7), followed by Mexico (n = 5), Iran (n = 3), Germany, Egypt, Saudi Arabia, Czech Republic, China (n = 2), United Kingdom, Turkey, Austria, New Zealand, and Finland (n = 1). Furthermore, the most widely used diagnostic test to evaluate *Toxoplasma* antibodies was the enzyme-linked immunosorbent assay (ELISA) method.

**Table 1:** Characteristics of the included studies for *T*. *gondii* infection in depressed patients and controls.

No	First author	Publication year	Place of study	Type of study	Method	Test	Results	Age (years ± SD)	Sex (N)
1	Conejero-Goldberg et al. [22]	2003	USA	Case control	Nested-PCR	-	Not significant	P: 39.9 C: 48.7	—
2	Thomas et al. [23]	2004	United Kingdom	Cross sectional	Eiken latex agglutination test, Dye test, IgM ELISA, IFA	IgG and IgM	Not significant	P: <70 C: <70	—
3	Alvarado-Esquivel et al. [24]	2006	Mexico	Case control	ELISA	IgG and IgM	Not significant	P: 43.7±13.8 C: 42± 20.2	C: (F:55, M:125)
4	Cetinkaya et al. [25]	2007	Turkey	Case control	ELISA	IgG and IgM	Not significant	P: 37.4 ± 01 C: 37.17 ± 11.54	P: (F:24, M:26) C: (F:27, M:23)
5	Hinze-Selch et al. [26]	2007	Germany	Case control	Toxo-Spot IF	IgG	Significant	P: 46.0 ±15.5 C: 38.9 ± 13.3	—
6	Hamidinejat et al.[27]	2010	Iran	Case control	ELISA	IgG and IgM	Not significant	P: 18–58 C: 18–58	—
7	Xiao et al.[28]	2010	China	Case control	ELISA	IgG	Not significant	P: 15–65 C: 15–65	—
8	Groër et al.[21]	2011	Austria	Cross sectional	ELISA	IgG	Significant	P: 18–45 C: 18–45	F:414
9	Pearce et al. [29]	2012	USA	Cross sectional	EIA	IgG	Significant	P: 15–39 C: —	
10	El-Sayed et al. [30]	2012	Egypt	Case control	ELISA	IgG	Not significant	P: 37 ± 11 C: 37.76 ± 10.50	P: (F:11, M:19) C: (F:12, M:18)
11	Gale et al.[31]	2014	USA	Cross sectional	ELISA	IgG	Not significant	29.7 ± 0.4	—
12	Markovitz et al.[32]	2015	USA	Cross sectional	ELISA	IgG	Not significant	P: ≥18 C: —	C: (F:217, M:139)
13	Al-Hussainy et al. [33]	2015	Saudi Arabia	Cross-sectional	ELISA	IgG and IgM	Not significant	P: ≥15 C: ≥15	P: (F:21, M:18)
14	Cong et al. [34]	2015	China	Case control	ELISA	IgG and IgM	Not significant	P: 16–91 C: 16–91	C: (F:238, M:207)
15	Wilking et al. [35]	2016	Germany	Cross sectional	ELFA	IgG	Not significant	P: 18–79 C: —	—
16	El-Aal et al. [36]	2016	Egypt	Case control	ELISA	IgG	Not significant	P: 2–46 C: 2–46	P: (F:73, M:45) C: (F:33, M:27)
17	Shiadeh et al. [37]	2016	Iran	Case control	ELISA	IgG	Not significant	P: 28.9 ± 5.6 C: 28.2 ± 5.4	F:116 F:244
18	Alvarado-Esquivel et al. [38]	2016a	Mexico	Case control	ELISA	IgG and IgM	Not significant	P: 38.65 ± 12.93 C: 38.66 ± 12.89	P: (F:64, M:25) C: (F:260, M:96)
19	Alvarado-Esquivel et al. [39]	2016b	Mexico	Case control	EIA	IgG and IgM	Significant	P: 39.43 ± 14.05 C: 39.45 ±13.98	P: (F:42, M:23) C: (F:168, M:92)
20	Gale et al.[40]	2016	USA	Cross sectional	EIA	IgG	Not significant	P: 20–80 C: —	—
21	Sugden et al. [41]	2016	New Zealand	Cross sectional	EIA	IgG	Not significant	P: 38 C: —	—
22	Zaki et al. [42]	2016	Saudi Arabia	Case control	ELISA	IgG and IgM	Not significant	P: 35.3±9.1 C: 34.7±8.9	C: (F:94, M:68)
23	Flegr and Escudero [43]	2016	Czech Republic	Cross sectional	CFT and ELISA	IgG and IgM	Not significant	P: M: 34.0±10.5, F: 36.5±12.3 C: M: 34·8±12·7, F: 32,4±11·0	P: (F:41, M:6) C: (F:356, M:853)
24	Alvarado-Esquivel et al. [44]	2017a	Mexico	Case control	ELISA and PCR	IgG and IgM	Not significant	P: 69.08±11.39 C: 68.56±10.08	C: (F:105, M:90)
25	Suvisaari et al. [45]	2017	Finland	Cross sectional	ELISA	IgG	Not significant	P: ≥30 C: —	—
26	Massa et al. [46]	2017	USA	Cross sectional	ELISA	IgG	Significant	P: 44.02±11.89 C: —	—
27	Abdollahian et al. [47]	2017	Iran	Case control	ELISA	IgG and IgM	Significant	P: 35±11.61 C: 38±13.2	C: (F:180, M:170)
28	Flegr and Horáček [48]	2017	Czech Republic	Cross sectional	CFT and ELISA	IgG and IgM	Not significant	M: 35.6±12.4 F: 32.9±12.3	—
29	Alvarado-Esquivel et al.[49]	2017b	Mexico	Case control	EIA and ELFA	IgG and IgM	Not significant	P: 23.40 ± 8.36 C: 23.01 ± 7.55	F:200 F:200
30	Yolken et al. [50]	2017	USA	Case control	ELISA	IgG	Not significant	P: 37.8 C:32.7	P: (F:40, M:24) C: (F:358, M:213)

ELISA: Enzyme-linked immunosorbent assay, IFA: Indirect immunofluorescence assay, CFT: Complement fixation test, EIA: Enzyme immunoassay, Nested-PCR: Nested-polymerase chain reaction, IgG: Immunoglobulin G, IgM: Immunoglobulin M, P: Pateint, C: Control, F: Female, M: Male, N: Number

**Table 2:** Characteristics of the included studies for *T*. *gondii* serological analysis in depressed patients and controls.

No	First author	N	Case: MDD^+^ (n)	Control: MDD^-^ (n)	MDD^+^ & T^+^ (n, %)	MDD^-^ & T^+^ (n, %)	OR (95% CI)	p-value
1	Conejero-Goldberg et al. [22]	29	3	26	0 (0%)	0 (0%)	7.57 (0.13–445.73)	—
2	Thomas et al. [23]	18	18	—	7 (38.9%)	—	0.81 (0.29–2.3)	—
3	Alvarado-Esquivel et al. [24]	183	3	180	1 (33.33%)	16 (8.9%)	5.13 (0.44–59.67)	*p* = 0.25
4	Cetinkaya et al. [25]	100	50	50	12 (24%)	11 (22%)	1.12 (0.44–2.84)	—
5	Hinze-Selch et al. [26]	679	465	214	221 (47.5%)	55 (25.70%)	2.62 (1.83–3.74)	—
6	Hamidinejat et al.[27]	94	46	48	15 (32.6%)	14 (29.2%)	1.18 (0.49–2.82)	—
7	Xiao et al. [28]	2663	29	2634	4 (13.8%)	329 (12.5%)	1.12 (0.39–3.24)	—
8	Groër et al. [21]	414	414	—	44 (10.63%)	—	—	—
9	Pearce et al. [29]	515	515	—	65 (12.62%)	—	0.73 (0.56–0.95)	*p*>0.05
10	El-Sayed et al. [30]	50	30	20	12 (40%)	6 (30%)	1.56 (0.47–5.18)	—
11	Gale et al. [31]	129	129	—	—	—	0.48 (0.19–1.26)	—
12	Markovitz et al. [32]	76	76	—	—	—	1.09 (0.57–2.10)	—
13	Al-Hussainy et al. [33]	94	39	55	10 (25.64%)	8 (14.55%)	2.03 (0.72–5.72)	*p* = 0.001
14	Cong et al. [34]	484	39	445	3 (7.7%)	55 (12.36%)	0.59 (0·18–1.98)	—
15	Wilking et al. [35]	6515	768	5,747	409 (53.25%)	3161 (55%)	0.93 (0·8–1.08)	….
16	El-Aal et al. [36]	178	118	60	24 (20.3%)	7 (11.7%)	1.93 (0.78–4.79)	—
17	Shiadeh et al. [37]	360	116	244	69 (59.5%)	125 (51.23%)	1.4 (0.89–2.19)	*p* = 0.142
18	Alvarado-Esquivel et al. [38]	363	7	356	0 (0%)	22 (6.2%)	0.99 (0.05–17.91)	—
19	Alvarado-Esquivel et al. [39]	325	65	260	15 (23.1%)	18 (6.9%)	4.03 (1.91–8.54)	*p*<0.001
20	Gale et al.[40]	1001	1001	—	—	—	0.83 (0.64, 1.06)	—
21	Sugden et al. [41]	127	127	—	35 (27.56)	—	1.01 (0.66–1.54)	—
22	Zaki et al. [42]	168	6	162	2 (33.3%)	24 (14.8%)	2.88 (0.66–16.58)	*p* = 0.387
23	Flegr and Escudero [43]	1256	47	1209	8 (17.02%)	285 (23.57%)	0.67 (0.31–1.44)	*p* = 0·109
24	Alvarado-Esquivel et al. [44]	230	35	195	6 (17.1%)	21 (10.8%)	1.71 (0.64–4.61)	*p* = 0.02
25	Suvisaari et al. [45]	5917	288	5629	70 (20.35%)	1331 (19.67%)	1.04 (0.8–1.37)	*p* = 0.75
26	Massa et al. [46]	153	153	—	—	—	0.23 (0.087–0.613)	—
27	Abdollahian et al. [47]	385	35	350	18 (51.43%)	120 (34.28%)	2.03 (1.01–4.08)	—
28	Flegr and Horáček [48]	78	78	—	—	—	1.38 (0.84–2.25)	*p* = 0.203
29	Alvarado-Esquivel et al. [49]	400	200	200	9 (4.5%)	12 (6.0%)	0.74 (0.3–1.79)	*p* = 0.50
30	Yolken et al.[50]	635	64	571	5 (7.8%)	35 (6.1%)	1.3 (0.49–3.44)	—

N and n: Number, CI: Confidence interval; MDD^+^: Individuals with depression; MDD^-^: Individuals without depression; MDD^+^ & T^+^: Individuals with depression and *Toxoplasma* positive; MDD^-^ & T^+^: Individuals without depression and *Toxoplasma* positive; OR: Odds ratio

## Discussion

This systematic review and meta-analysis aimed to quantify the pooled ORs of anti-*T*. *gondii* IgG and IgM antibodies in depressed patients and compare them with those of control groups. The results obtained from the current study showed that the overall ORs of anti-*T*. *gondii* IgG and IgM antibodies in patients with MDD were 1.15 (95% CI: 0.95–1.39) and 1.69 (95% CI: 0.72–3.96), respectively. Based on these results, toxoplasmosis is not considered a risk factor for depressed patients. There are several reasons accounting for these negative findings: 1) infectious agent does not play an etiologic role in major depression, 2) the infectious agent has attended earlier in individuals’ life but are no longer detectable [22].

Based on the results of the meta-analysis, the depressed patients had a lower seroprevalence of *T*. *gondii* as compared to the controls. Major depression showed no association with anti-*Toxoplasma* IgG and anti-*Toxoplasma* IgM. Our results are inconsistent with those of the systematic reviews evaluating the association between toxoplasmosis and psychiatric disorders, including epilepsy (OR = 2.25) [11], bipolar disorder (OR = 1.26) [8], obsessive-compulsive disorder (OR = 1.96) [51], schizophrenia (OR = 1.81) [9], and addiction (OR = 1.91) [9].

On the other hand, our results are in line with those of a couple of previous meta-analyses that showed no association between *T*. *gondii* infection and depression [9, 15]. Wang *et al*. in 2014 [15] evaluated the relationship between different infectious agents and depression. In this meta-analysis, only three studies had addressed the relationship between *T*. *gondii* infection and depression. After analyzing various studies, no significant association was found, and the OR was calculated as 1.36. All three studies were included in the current meta-analysis.

In a unique meta-analysis published in 2015 [9], Sutterland *et al*. assessed the prevalence of *T*. *gondii* infection in several psychiatric disorders using both published and unpublished studies. They obtained a non-significant OR of 1.2 indicating no general difference between healthy subjects and depressed patients in terms of *T*. *gondii* infection prevalence. Since the present study only included the published studies, 7 out of the 9 studies reviewed in the mentioned study were investigated in the current meta-analysis. One of the two non-investigated studies was unpublished, and the other study was a summary of the paper presented at a congress [52]. In addition, 22 studies were not investigated in the mentioned meta-analysis; however, they were included in the current study due to meeting our inclusion and exclusion criteria.

The advantages of this meta-analysis are as follows: 1) the investigation of a larger number of cases and controls, compared to those of the previous studies that improves the statistical power to assess the association between toxoplasmosis and depression, 2) inclusion of studies from eight other countries in the meta-analysis that intensifies the consistency of the association, 3) analysis of case-control and cross-sectional studies based on OR, and 4) exclusive attention to MDD.

According to Fig 2, the lowest and highest ORs are related to the studies performed by Massa *et al*. [46] and Conejero-Goldberg *et al*. [22], respectively. This difference could be due to the sole use of postmortem brain samples by Conejero-Goldberg *et al*. [22]. In addition, the quality of the mentioned study was low, and the number of the subjects was small. However, Massa *et al*. used a larger sample size with a different set of psychiatric diagnoses. Moreover, race, educational level, and economic status can result in different ORs [46].

Difference in the sensitivity and specificity of detection methods, different geographic regions, age, gender, and ethnic groups are the main reasons for the difference in the prevalence of *T*. *gondii* [6, 9, 53]. Given the incomplete data of the studies assessing the relationship between different variables (e.g., age, gender, ethnic, diet, family history, parasite strain, socioeconomic level) and the prevalence of toxoplasmosis, meta-analysis was not applicable for these variables. Furthermore, due to the lack of the evaluation of these risk factors in some studies, these variables cannot be analyzed; accordingly, this is considered as a basic gap.

For example, the gender agent has an apparent impact on the psychomotor performance of humans [54]. Female sex hormones are known to manipulate dopaminergic actions in some parts of the brain. The occurrence of degenerative disorders in women, such as Parkinsonism, decreases the protective effect of dopamine [55]. Conversely, higher levels of testosterone in men can cause toxoplasmosis associated with changes in human and animal behaviors [56]. Therefore, this variable is important in evaluating the relationship between depression and *T*. *gondii* and needs to be addressed.

The possible mechanism underlying the behavioral changes correlated with latent toxoplasmosis is the presence and spread of *T*. *gondii* cysts in the central nervous system. Berenreiterová *et al*. [57] reported that *Toxoplasma* latent cysts in mice were distributed all over the brain; it would seem possible that parasitic cysts in these areas in humans could alter both frontal and limbic regions which could then result in behavioral and emotional changes. Tryptophan catabolic shunt and serotonin during reactivation stage of *T*. *gondii* infection may contribute to the development of depressive-like behavior [58]. In addition, neuronal function and immune-mediated dopamine and serotonin synthesis may be the mechanism of contribution of *T*. *gondii* infection to behavioral disorders [59]. The production of a large amount of dopamine by parasite increases the destruction of the cyst walls and production of tachyzoites [60]. In a study, the treatment of rats with latent toxoplasmosis by means of a dopamine-2 antagonist resulted in the reduction of their risky behaviors [61].

T helper 1 (Th1), natural killer cells, and nitric oxide interfere in host response to *T*. *gondii* infection. The Th1 cells secrete interferon-γ (IFN-γ) and pro-inflammatory cytokines that are important in impeding tachyzoite replication and preventing the reactivation of the tissue cysts of the central nervous system [62]. Inflammatory cytokines, such as IFN-c, tumor necrosis factor-a, and interleukin 6, are parts of the inflammatory response to *T*. *gondii*, leading to the production of glucocorticoids, which affects neuroplasticity [63]. Reduced serotonin production and tryptophan depletion in the brain may contribute to the incidence of depression caused by such cytokines as IFN-γ that could lead to the activation of indoleamine 2, 3-dioxygenase [64, 65]. Activation of guanosine-triphosphate-cyclohydrolase-1 by IFN-γ and other pro-inflammatory cytokines (e.g., IFN-α and IFN-β) increases the production of neopterin, enhances nitrate concentration through the production of tetrahydrobiopterin, and decreases phenylalanine and tyrosine levels. Amine acid phenylalanine has a key role in the biosynthesis of norepinephrine and dopamine. Sleep disturbance, fatigue, and disorders of the gastrointestinal and musculoskeletal systems are associated with decreased dopamine synthesis [64]. In a study, it was reported that the signs and symptoms of depression were ameliorated after the treatment of *T*. *gondii* infection although patient was unresponsive to the conventional anti-depressants [66].

### Limitations

There are several restrictions in our research. One of these limitations was the adoption of different sampling techniques, such as Facebook-based snowball method, in various studies. Investigation of different age groups in different studies was another limitation since individuals with a longer duration of *Toxoplasma* seropositivity could have a higher level of depression. The type of studies reviewed is also a limitation of this study as the nature of cross-sectional study did not allow us to investigate possible causal relationships between *T*. *gondii* and depression. Also, in most case-control studies, the case and control groups were matched for age and sex, but cross-sectional studies cannot be matched for age and sex and therefore may be one of the reasons for the difference in the significance level of studies. In some cross sectional studies, despite having a large population-based study sample, the number of participants with depression was still limited, and the lack of significant association of *T*. *gondii* with depression despite higher prevalence in seropositivity and higher serointensity may reflect limited statistical power. Another limitation was that most of the included studies did not have sufficient information on disease status or severity. In addition, the studies had variable quality. Furthermore, a few studies were conducted on limited population, such as pregnant women; therefore, the results cannot be extrapolated to the general population. As the final limitation, the reviewed studies did not evaluate the effect of *Toxoplasma* strains on depression.

## Conclusions

Based on the results of this meta-analysis, no statistically significant association was observed between toxoplasmosis and MDD. However, according to the results of the reviewed studies, especially those of the case-control studies, the potential role of toxoplasmosis in depression cannot be completely ruled out. Therefore, it is necessary to perform further research to clarify the role of *T*. *gondii* exposure in the clinical characteristics of MDD and determine the detailed association between *T*. *gondii* and dysthymia or mild and moderate depression. In addition, it is recommended to evaluate the effect of the antibody titers on the association between depression and *Toxoplasma*.

## Supporting information

S1 Table PRISMA 2009 checklist. (DOC)

S2 Table NOS checklist. (DOCX)
