# Supplementary material for: Is there any association between Toxoplasma gondii infection and depression? A systematic review and meta-analysis
Source: PLoS One. 2019 Jun 13;14(6):e0218524. doi: 10.1371/journal.pone.0218524 (PMC6564815; doi:10.1371/journal.pone.0218524)
Supplement: S2 Table — (DOCX) [file pone.0218524.s002.docx]

**S2 Table. Quality assessment of included studies based on the Newcastle-Ottawa Scale (NOS)**

| Author, (ref) | Type of study | Selection | Comparability | Outcome | Total (score) |
| --- | --- | --- | --- | --- | --- |
| (Conejero-Goldberg *et al.*, 2003) | case-control | 3 | 0 | 3 | 6 |
| (Thomas *et al.*, 2004) | cross sectional | 3 | 2 | 2 | 7 |
| (Alvarado-Esquivel *et al.*, 2006) | case-control | 3 | 2 | 3 | 8 |
| (Cetinkaya *et al.*, 2007) | case-control | 3 | 2 | 3 | 8 |
| (Hinze-Selch *et al.*, 2007) | case-control | 2 | 2 | 2 | 6 |
| (Hamidinejat *et al.*, 2010) | case-control | 1 | 0 | 3 | 4 |
| (Xio *et al.*, 2010) | case-control | 3 | 1 | 3 | 7 |
| (Groër *et al.*, 2011) | cross sectional | 2 | 2 | 2 | 6 |
| (Pearce *et al.*, 2012) | cross sectional | 3 | 2 | 2 | 7 |
| (El-Sayed *et al.*, 2012) | case-control | 4 | 2 | 3 | 9 |
| (Gale *et al.*, 2014) | cross sectional | 3 | 2 | 2 | 7 |
| (Markovitz *et al.*, 2015) | cross sectional | 3 | 2 | 2 | 7 |
| (Al-Hussainy *et al.*, 2015) | case-control | 3 | 2 | 2 | 7 |
| (Cong *et al.*, 2015) | case-control | 2 | 2 | 3 | 7 |
| (Wilking *et al.*, 2015) | cross sectional | 2 | 2 | 2 | 6 |
| (El-Aal *et al.*, 2016) | case-control | 3 | 0 | 3 | 6 |
| (Shiadeh *et al.*, 2016) | case-control | 4 | 2 | 3 | 9 |
| (Alvarado-Esquivel *et al.*, 2016 a) | case-control | 3 | 2 | 3 | 8 |
| (Alvarado-Esquivel *et al.*, 2016 b) | case-control | 3 | 2 | 3 | 8 |
| (Gale *et al.*, 2016) | cross sectional | 2 | 2 | 2 | 6 |
| (Sugden *et al.*, 2016) | cross sectional | 2 | 1 | 2 | 5 |
| (Zaki WM *et al.*, 2016) | case-control | 3 | 2 | 3 | 8 |
| (Flegr *et al.*, 2016) | cross sectional | 2 | 0 | 2 | 4 |
| (Alvarado-Esquivel *et al.*, 2017) | case-control | 3 | 2 | 3 | 8 |
| (Suvisaari *et al.*, 2017) | cross sectional | 3 | 2 | 2 | 7 |
| (Massa *et al.*, 2017) | cross sectional | 2 | 2 | 2 | 6 |
| (Abdollahian *et al.*, 2017) | case-control | 2 | 2 | 3 | 7 |
| (Flegr and Horáček, 2017)  (Alvarado-Esquivel *et al.*, 2017)  (Yolken *et al.*, 2017) | cross sectional  case-control  case-control | 1  3  2 | 2  2  0 | 2  3  3 | 5  8  5 |

High quality (7 – 9), Moderate quality (4 – 6) or Low quality (≤3) in case-control studies and High quality (6 and 7), Moderate quality (3 – 5) or Low quality (1 and 2) in cross sectional studies.
